# Monitoring Aqueous Sucrose Solutions Using Droplet Microfluidics: Ice Nucleation, Growth, Glass Transition, and Melting

**DOI:** 10.1021/acs.langmuir.3c03798

**Published:** 2024-03-18

**Authors:** Leif-Thore Deck, Nadia Shardt, Imad El-Bakouri, Florin N. Isenrich, Claudia Marcolli, Andrew J. deMello, Marco Mazzotti

**Affiliations:** ∇ Institute of Energy and Process Engineering, ETH Zurich, Zurich 8092, Switzerland; ‡ Institute for Atmospheric and Climate Science, ETH Zurich, Zurich 8092, Switzerland; ¶ Department of Chemical Engineering, Norwegian University of Science and Technology (NTNU), Trondheim 7491, Norway; § Institute for Chemical and Bioengineering, ETH Zurich, Zurich 8092, Switzerland

## Abstract

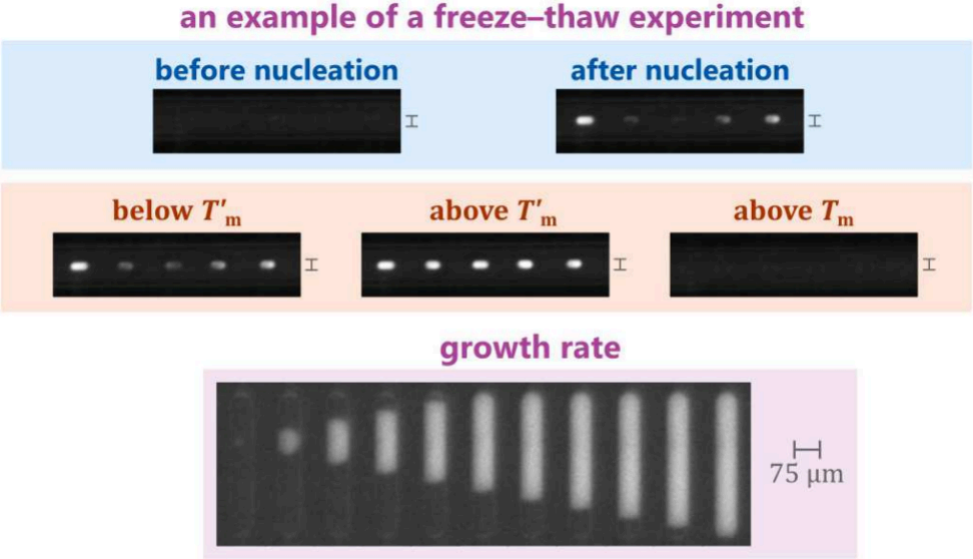

Freezing and freeze-drying processes are commonly used to extend the shelf life of drug products and to ensure their safety and efficacy upon use. When designing a freezing process, it is beneficial to characterize multiple physicochemical properties of the formulation, such as nucleation rate, crystal growth rate, temperature and concentration of the maximally freeze-concentrated solution, and melting point. Differential scanning calorimetry has predominantly been used in this context but does have practical limitations and is unable to quantify the kinetics of crystal growth and nucleation. In this work, we introduce a microfluidic technique capable of quantifying the properties of interest and use it to investigate aqueous sucrose solutions of varying concentration. Three freeze–thaw cycles were performed on droplets with 75-μm diameters at cooling and warming rates of 1 °C/min. During each cycle, the visual appearance of the droplets was optically monitored as they experienced nucleation, crystal growth, formation of the maximally freeze-concentrated solution, and melting. Nucleation and crystal growth manifested as increases in droplet brightness during the cooling phase. Heating was associated with a further increase as the temperature associated with the maximally freeze-concentrated solution was approached. Heating beyond the melting point corresponded to a decrease in brightness. Comparison with the literature confirmed the accuracy of the new technique while offering new visual data on the maximally freeze-concentrated solution. Thus, the microfluidic technique presented here may serve as a complement to differential scanning calorimetry in the context of freezing and freeze-drying. In the future, it could be applied to a plethora of mixtures that undergo such processing, whether in pharmaceutics, food production, or beyond.

## Introduction

The freezing behavior of aqueous solutions is of broad interest to multiple disciplines, ranging from the atmospheric sciences^1−7^ and cryobiology^8−13^ to the manufacturing of pharmaceuticals^14,15^ and food.^16,17^ In all these fields, it is important to measure and predict the equilibrium state expected at certain conditions (temperature, pressure, composition, etc.) and to assimilate this information in the form of a phase diagram.^18^ Due to the energy barrier required to form an ice nucleus,^19,20^ metastable supercooled water may persist for prolonged periods of time under relevant conditions, e.g., in cloud droplets in the atmosphere^4,21,22^ or in vials filled with ultrapure, particulate-free aqueous solutions of biopharmaceutical formulations.^23,24^ The temperature at which nucleation is actually observed depends on parameters such as the volume of the bulk solution, the cooling rate, and the mode of nucleation (whether homogeneous or heterogeneous). Thus, knowledge about the relevant kinetic parameters is required, including the rates at which ice crystals nucleate and grow, to understand and design processes of relevance to, for example, the pharmaceutical industry.

For the storage and distribution of biopharmaceuticals, processes such as freezing and freeze-drying are used to remove water from the active ingredients and extend the shelf life of the drug product.^14,15^ Designing such processes necessitates detailed knowledge of how the solution containing the active ingredients undergoes phase transitions. For instance, the size and morphology of the ice crystals that form influences the drying rate and, in the end, the physical characteristics of the freeze-dried formulation.^20,23,25,26^ Empirical guidelines suggest that larger ice crystals enable faster drying times and that higher nucleation temperatures are correlated to larger crystals.^23,25^ Hence, process conditions are chosen to promote higher nucleation temperatures, e.g., by using slow cooling rates.^14,15^ After freezing is completed, the drying stage in freeze-drying must be designed such that the frozen drug product remains below a certain critical temperature, so as to avoid the collapse of its delicate microstructure.^14,15^

Differential scanning calorimetry (DSC) is used to detect key thermal events (and temperatures) across the liquid–solid phase diagram. That is, the interpretation of heat flow to/from a sample using DSC permits the identification of phase changes, including glass transitions. It is common practice in the freeze-drying literature to link the critical temperature of collapse to a glass transition temperature.^14,15,20^ For some solutions, such as those containing proteins,^27^ the accurate identification of glass transitions can be practically challenging due to weak signal strength.^28,29^ For this reason, alternative technologies such as freeze-dry microscopy have been developed which enable the screening of process conditions for collapse phenomena in a microscopic sample.^30,31^ However, neither DSC nor freeze-dry microscopy allows for the measurement of kinetic parameters such as nucleation rate and crystal growth rate.

Herein, we demonstrate the use of microdroplets to access important phase transitions and temperatures on the isobaric phase diagram of sucrose–water mixtures that have not otherwise been possible to quantify in bulk volumes (μL–mL). Specifically, populations of microdroplets were generated in a microfluidic device and stored in perfluoroalkoxy alkane (PFA) tubing using an instrument named the Microfluidic Ice Nuclei Counter Zurich (MINCZ).^32,33^ The droplets then were cooled and warmed, during which temporal changes in droplet brightness were observed, corresponding to changes in the phase(s) present. This experimental tool could be used in the future for other mixtures that undergo detectable changes in brightness as the temperature range between freezing and melting is traversed. In addition, for highly concentrated sucrose solutions, we quantified the linear crystal growth rate at significantly lower temperatures than previously reported.^34^ Thus, both the thermodynamic and kinetic properties of a solution could be determined in a single experimental setup.

## Qualitative Trends in Droplet Brightness

**Figure 1:**
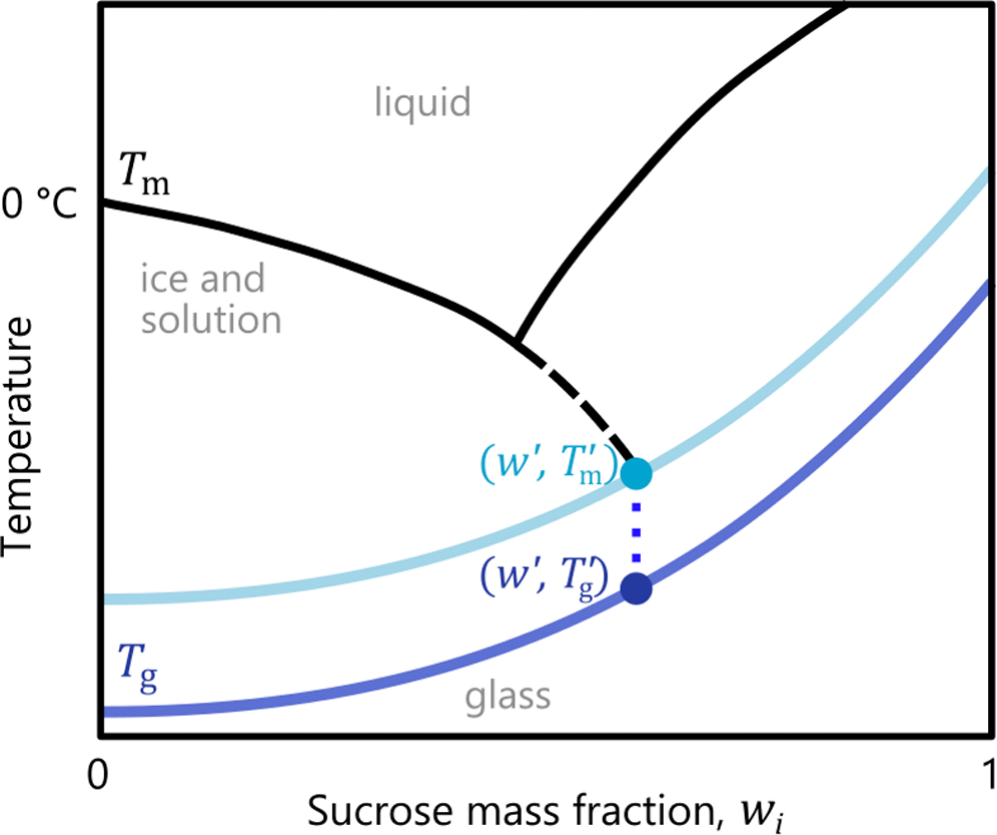
A qualitative, extended phase diagram of sucrose–water mixtures showing the melting temperature *T*_m_ (solid black line), the composition-dependent glass transition temperature *T*_g_ (solid dark blue line), and the temperature of the maximally freeze-concentrated solution *T*_m_^′^ with corresponding composition *w*′ (light blue dot). The point (*w*′, *T*_m_^′^) lies on an iso-viscosity line (solid light blue line) at which the viscosity is sufficiently high to arrest further ice growth from the highly concentrated solution. The glass transition temperature at composition *w*′ is denoted by *T*_g_^′^ (dark blue dot).

Figure 1 outlines a qualitative extended phase diagram as a function of temperature and sucrose mass fraction. It is an *extended* phase diagram because it shows features in addition to those derived from equilibrium thermodynamics that are relevant to the freezing process. First, the equilibrium melting line is extended beyond the eutectic concentration (shown as a dashed line), where the solution is supersaturated with respect to the solute, sucrose. This is because during freezing, pure ice crystals are formed, which increases the solute concentration in the unfrozen solution. This increase continues beyond the eutectic point, at which point crystallization of the solute may eventually occur. Some solutes such as mannitol commonly nucleate during the freezing process.^14,35^ For sucrose, however, such behavior has neither been reported nor observed here.

Figure 1 also features two pieces of information about glass transitions. The first is the glass transition temperature of the metastable solution in which neither ice nor sucrose has nucleated. This temperature depends on solution composition, and it is referred to as *T*_g_. The second is a point highlighted by the coordinates (*w*′, *T*_g_^′^), which represents the glass transition temperature of the maximally freeze-concentrated solution at a sucrose mass fraction of *w*′. As a result of this concentration process, the viscosity of the solution gradually increases until it becomes high enough to inhibit further crystal growth.

While *T*_g_ is associated with the ultrahigh viscosity of a glassy state (10^12^ Pa s),^36^ there is a higher temperature where ice growth in the sucrose–water freeze-concentrate is inhibited—at a lower viscosity (approximately 10^8^ Pa s),^36^ shown by the light blue *iso-viscosity line* in Figure 1. Qualitatively, this iso-viscosity line is a vertical translation of the *T*_g_ curve. The point (*w*′, *T*_m_^′^) is the intersection of the iso-viscosity line and the melting line. *w*′ represents the highest concentration level attainable in the freeze-concentrate, and the solution with this concentration is referred to as *maximally freeze-concentrated*. The value of *w*′ is independent of the solution’s initial composition, which only determines the relative amounts of the two phases that form upon freezing, i.e., the pure ice crystals and the freeze-concentrate. Both the physical interpretation and the name of the temperature associated with the point (*w*′, *T*_m_^′^) are inconsistently used in the literature (see Sacha and Nail^37^ for a comprehensive discussion), with the terms *glass transition temperature of the freeze-concentrate*(27,38) and *antemelting temperature*(39) both in use.

The maximally freeze-concentrated solution (*w*′, *T*_m_^′^) is of immediate interest to the freezing and freeze-drying of biopharmaceuticals, whereas *T*_g_^′^ is not. This is because the primary drying stage of the freeze-drying process must be designed such that the frozen product remains at a temperature below a critical value (termed *T*_c_). A large number of studies have revealed that for formulations where the solutes do not crystallize during freezing (as is the case for sucrose-based formulations) this critical temperature lies close to the value of *T*_m_^′^, so that the measurement of *T*_m_^′^ has become a standard practice in the field.^15,20,27,40^ The associated concentration *w*′ is of relevance to the storage of frozen biopharmaceutical drug products, as it governs the long-term stability of the active ingredients in the freeze-concentrated solution.^40,41^ For the case of sucrose solutions, *T*_m_^′^ is reported to lie at about −33 °C independent of concentration, whereas *T*_g_^′^ lies between −49 °C and −45 °C, as measured by DSC.^27,37^ While DSC can be used to extract both temperatures,^39,40^ the microfluidic technique introduced here allows for the measurement of *T*_m_^′^ only. As noted by Sacha and Nail,^37^ calorimetric investigations of these temperatures have been interpreted inconsistently across research groups, identifying a need for visual data, which we provide below, to aid in interpretation.

**Figure 2:**
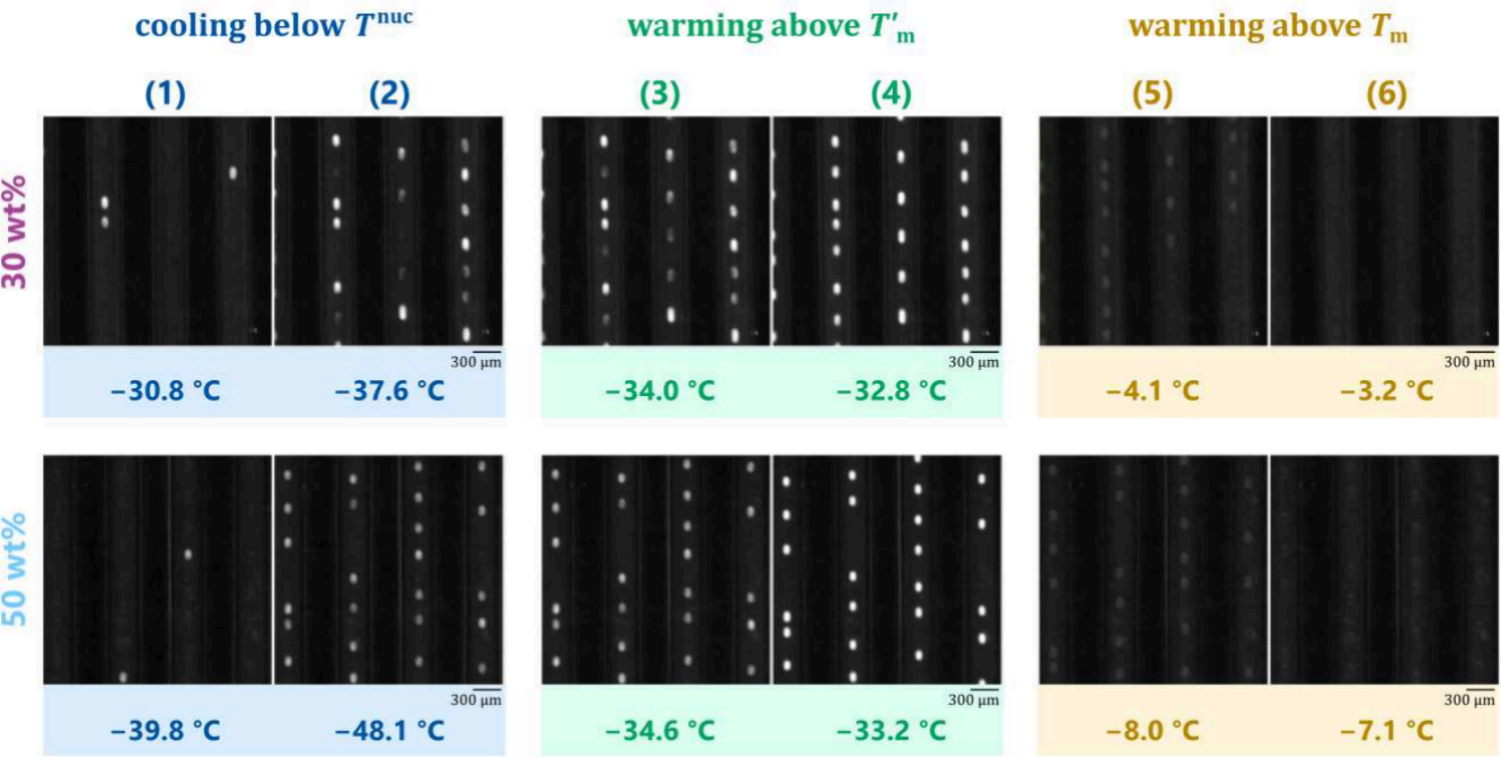
Sequence of images during the first cycle of the series depicted in Figures 3 and 5 (▲) for the 30 wt % (*T*_m_ = −3.1 °C) and 50 wt % (*T*_m_ = –7.2 °C) sucrose mixtures corresponding to the processes depicted by arrows in the schematic on the left. (1) to (2) shows the progression of droplet freezing as temperature is decreased below the droplet nucleation temperature *T*^nuc^; (3) to (4) shows the onset and end of the region associated with *T*_m_^′^ that lies at about −33 °C; and (5) to (6) shows the onset and end of melting (*T*_m_). All images are cropped areas of size 2.4 × 2.4 mm^2^ at the center of the full image. Onsets and ends of the *T*_m_^′^ and *T*_m_ region are the temperatures at which vertical bars are drawn in Figure 5(b).

Phase transitions in droplets upon temperature change can be observed optically by changes in how the droplet interacts with light, as illustrated in Figure 2 for droplets of two aqueous sucrose concentrations (30 wt % upper row and 50 wt % lower row). Aqueous sucrose solutions are optically transparent to visible light, as seen for the majority of droplets in panel (1). When ice formation occurs, the crystals reflect and scatter light, increasing the brightness of the phase against the dark background (bright spots in column (1)). The temperature at which ice is first detected is designated as the nucleation temperature, *T*^nuc^, and it varies among droplets due to the stochastic nature of nucleation. Upon further cooling, ice forms in more and more droplets, as can be seen in column (2), and intriguingly, frozen droplets differ in their brightness. As we will see later, this effect is related to the temperature at which nucleation takes place. After reaching the predefined minimum temperature, droplets were heated back to the initial temperature, as shown in columns (3) to (6). As long as the droplet temperature remained significantly below *T*_m_^′^ (around −33 °C, column (3)), droplet brightness did not change. As *T* approached *T*_m_^′^ (column (4)), the droplet brightness visibly increased, with all droplets eventually exhibiting a similar level of brightness.

Upon further heating, the temperature eventually approaches the melting point where the brightness of frozen droplets decreased (columns (5) to (6)) as the proportion of ice decreased. The pixel intensity continued to decrease until the melting point was surpassed, at which time fully liquid droplets were again observed (column (6)). Experiments were conducted for multiple sucrose concentrations between 1 wt % and 60 wt %, hence covering the entire concentration range below the eutectic composition.

## Quantitative Analysis of Freezing

### Nucleation Temperatures

Nucleation is a stochastic process;^19,42^ hence, a quantitative analysis of a solution’s nucleation behavior requires the measurement of many nucleation events. Given that only a single nucleation event takes place in each droplet (see the section on Ice Crystal Growth), many droplets must be monitored to generate statistically relevant nucleation data sets. We evaluate such data by computing the cumulative distribution function, i.e., the fraction of droplets frozen as a function of time over the course of an experiment. Since the thermal evolution during the experiment is recorded, it is possible to express such a distribution in terms of temperature.

**Figure 3:**
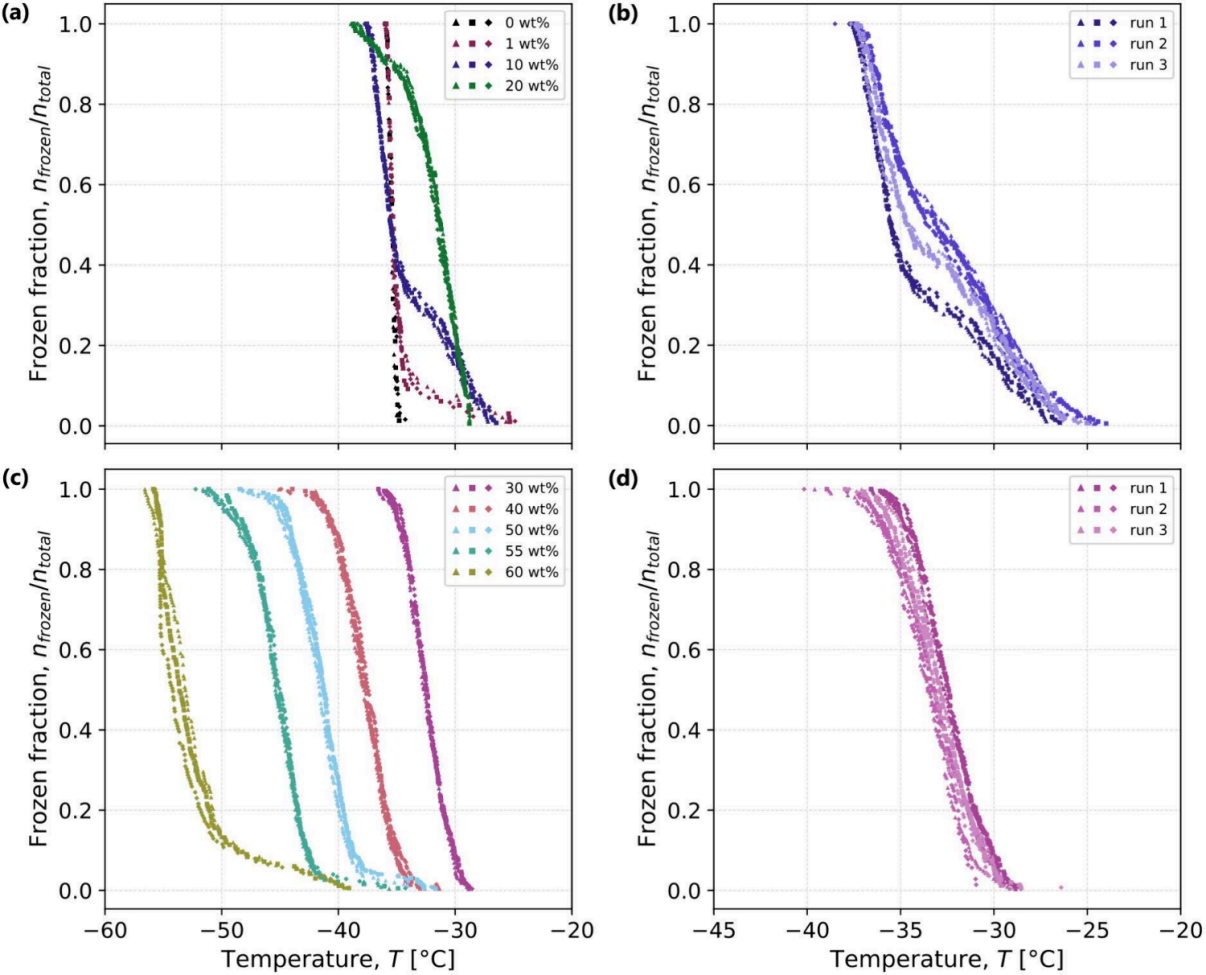
Frozen fraction of droplets (*n*_frozen_/*n*_total_) as a function of temperature and sucrose concentration observed in MINCZ. In panel (a), one droplet population (75-μm diameters) was generated from a stock solution at each listed concentration and underwent three consecutive freeze–thaw cycles at a rate of 1 °C  min^–1^. Each cycle is depicted with a different symbol (cycle 1: ▲; 2: ■; 3: ◆). The thermocouple accuracy is estimated to be ±0.2 °C.^32^ In panels (b) and (d), results from (a) and (b) are repeated for concentrations of 10 wt % and 30 wt % (labeled as run 1) with results from two additional independent droplet populations undergoing three freeze–thaw cycles (labeled as runs 2 and 3).

In general, the addition of a solute is expected to lower the temperature at which nucleation occurs. This is because a solute lowers water activity, and hence lower absolute temperatures are required to achieve the same value of the thermodynamic driving force for ice nucleation to occur.^1,19,43^ We have studied this effect recently for aqueous solutions containing sucrose, trehalose, and sodium chloride at different concentrations on the milliliter-scale,^44^ and here we aim to assess how such an effect manifests at the microscale. Figure 3 depicts the cumulative fraction of droplets frozen at each temperature for each studied sucrose concentration, summarized in two panels ((a) and (c)) to facilitate visual analysis. As was observed for the larger volumes in our earlier study,^44^ nucleation temperatures decrease with increasing concentration for sucrose concentrations above 20 wt %, as shown in panel (c).

For the lower concentrations shown in panel (a), namely 1 wt %, 10 wt %, and 20 wt %, the nucleation temperatures partially lie above those measured for pure water, contrary to expectation. All three distributions are bimodal; i.e., they exhibit a turning point (an abrupt change in the slope of the frozen fraction), at a frozen fraction of about 10% for the 1 wt % solution, at 30% for the 10 wt % solution and at about 90% for the 20 wt % solution. The fraction at which the turning point is positioned is reproducible across the three freeze–thaw cycles (different symbols) for all concentrations. In addition, Figure 3(b) shows that for three independent droplet populations each undergoing freeze–thaw cycles, the turning point also remains at the same temperature, and only the fraction of droplets freezing varies. At the higher concentration levels shown in panel (c), in contrast, distributions are generally unimodal, with only slight variation in nucleation temperatures between different droplet populations and between consecutive freeze–thaw cycles within the same droplet population. At the highest studied concentration (60 wt %), the distribution becomes bimodal again, with almost 10% of droplets freezing prior to the steepest increase in frozen fraction.

We propose the following explanation for these observations based on the concept that nucleation may occur either homogeneously (i.e., in the bulk volume) or heterogeneously (i.e., on surfaces of, e.g., impurities). The experiment for pure water is considered to feature homogeneous nucleation, supported by the fact that a large number of studies have observed similar nucleation temperatures for micrometer-sized droplets of ultrapure water in different setups.^45,46^ The increase in nucleation temperature in sucrose solutions with low concentration levels compared to pure water hence must be due to the presence of heterogeneous nucleation sites. If the number of heterogeneous nucleation sites is small, they are randomly distributed across droplets, and some droplets are expected to contain none; i.e., nucleation is homogeneous. This therefore explains the bimodal shape of the distributions. Given that the only difference between the experiments involving pure water and those involving sucrose solutions is the presence of sucrose, one must conclude that the heterogeneous nucleation sites are located on impurity particles present in the sucrose used in the experiments. Hence, a higher sucrose concentration implies that more nucleation sites are present, so that eventually heterogeneous nucleation takes place in virtually all droplets for concentrations of 30 wt % and higher. Similarly, the bimodal distribution of the 60 wt % solution may be due to heterogeneous nucleation sites not present at the lower concentrations. To confirm this conjecture regarding the 60 wt % solution, experiments at higher concentration levels are required, which were not carried out due to the experimental challenges in dealing with highly viscous solutions. An experimental approach to directly investigate whether heterogeneous nucleation is present would be to measure nucleation temperatures in droplets of different sizes, since smaller droplets are less likely to contain an impurity particle on which nucleation sites may be located. Doing so is indeed feasible with the microfluidic setup used here,^32^ but outside the scope of the work. Considering the literature, Miyata and Kanno^47^ reported only homogeneous nucleation of sucrose solutions using an emulsion-based DSC, in which the emulsion comprises microdroplets with diameters of a few μm,^48^ i.e., volumes more than 3 orders of magnitude smaller than the droplets measured here.

The presence of heterogeneous nucleation further explains the broadness of the measured nucleation temperature distributions on the order of 10 K, which is significantly wider than those for pure water of less than 3 K.^32^ Heterogeneous nucleation sites vary in the characteristic temperature at which they trigger nucleation,^49^ leading to a droplet-to-droplet variability in nucleation temperature in addition to the inherent stochasticity. Such additional variability has been widely reported in the literature, both in microdroplets^4,21,50^ and at the milliliter scale.^44,51,52^ Its quantitative study would require knowledge of droplet-specific mean nucleation temperatures, obtained by consecutively measuring nucleation temperatures for individual droplets over multiple freeze–thaw cycles. Given that our experiments comprise only three freeze–thaw cycles, we refrain from doing such an analysis; however, we point out that the setup presented here may indeed be capable of carrying out experiments with additional freeze–thaw cycles dedicated to the study of droplet-to-droplet variability.

Finally, it is worth comparing the monitored nucleation temperatures with those measured previously in vials filled with 1 mL sucrose solution prepared under the same condition.^44,51^ In these studies, nucleation was found to take place at an average supercooling of 13 K, with no significant dependence on sucrose concentration. That nucleation in the microdroplets takes place at significantly lower temperatures is explained by their smaller volume, which implies that each individual droplet contains only a few or no impurity particles, as discussed before, whereas volumes at the scale of vials contain many more impurities.

### Interplay between Nucleation Temperature and the Freeze-Concentrate

**Figure 4:**
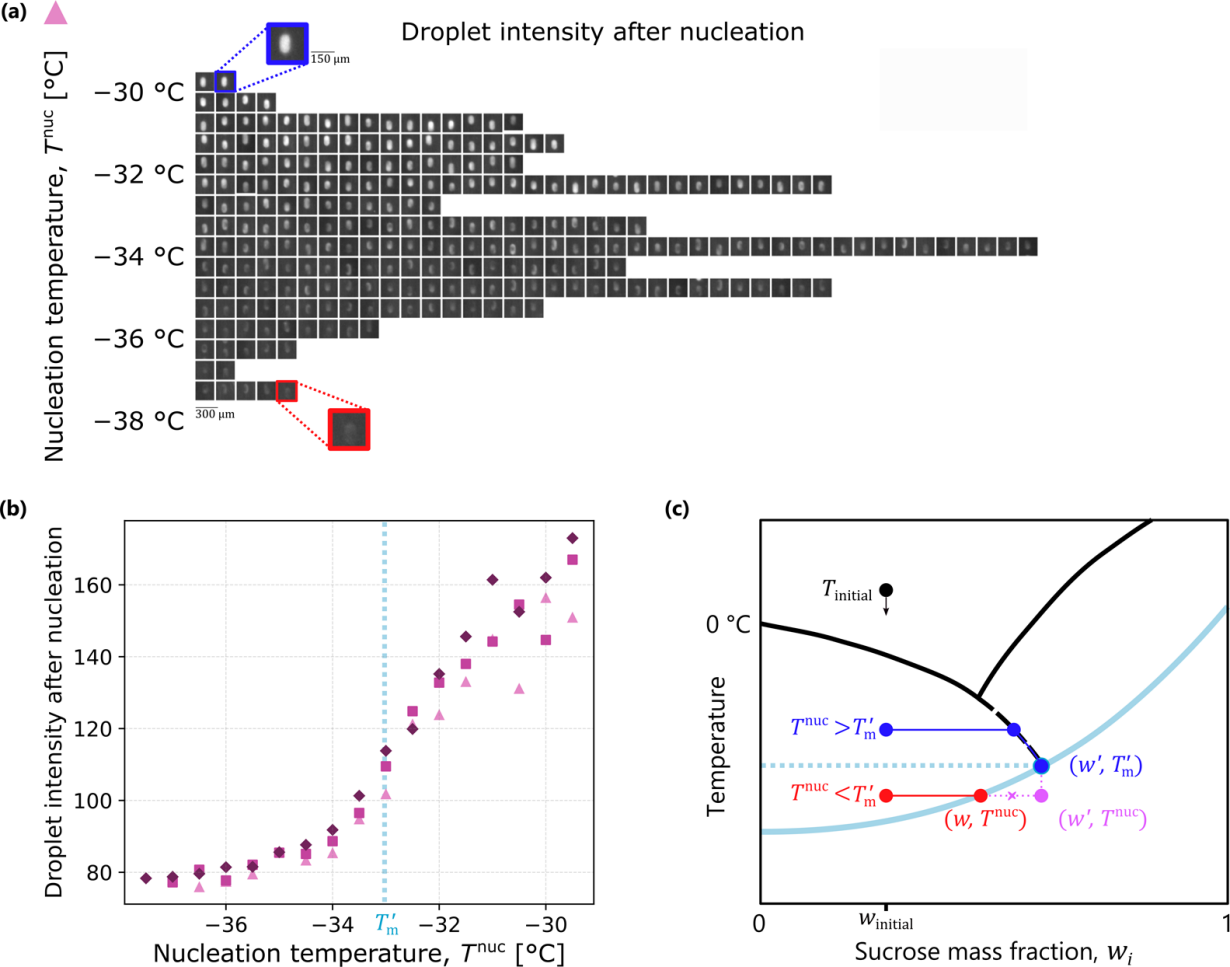
(a) Cropped images of frozen droplets with a sucrose concentration of 30 wt % as a function of their nucleation temperature binned in intervals of 0.5 °C for the first freeze–thaw cycle shown in Figure 3(c). (b) Pixel intensity (monochrome scale between 0 (black) and 255 (white)) of a circle with 9-pixel radius at the center of each identified droplet averaged over all droplets in the same bin for all three freeze–thaw cycles of 30 wt % droplets (cycle 1: ▲; 2: ■; 3: ◆) in Figure 3(c). (c) Schematic phase diagram illustrating the hypothesis for the change in droplet intensity when *T*^nuc^ < *T*_m_^′^: it is not possible to reach (*w*′, *T*^nuc^), because as ice grows, the iso-viscosity line is intersected at a lower concentration, and further ice growth ceases. *T*_m_^′^ is obtained from the analysis shown in Figures 5 and 6.

The visual appearance of the droplets after nucleation warrants further study, as their brightness was found to depend on the nucleation temperature. As an example, Figure 4(a) shows a cropped image of each droplet after nucleation as a function of temperature, binned in intervals of 0.5 °C for the first freezing ramp of the 30 wt % sucrose droplets. Qualitatively, the pixel intensity of droplets that nucleated at higher temperatures was significantly greater than those that nucleated at lower temperatures. Quantitatively, Figure 4(b) shows that the ratio between the intensities of the brightest and dimmest droplets is on the order of a factor of 2. Let us further recall from Figure 2 that upon reheating all droplets brighten to the same value of the pixel intensity. Physically, this behavior is due to the interplay between the temperature of ice nucleation (and the ensuing crystal growth) and whether the maximally freeze-concentrated solution is attained.

To elucidate this effect, one must consider the physical processes that take place during freezing within the droplets. We recall that the point (*w*′, *T*_m_^′^) is defined by the magnitude of viscosity that inhibits the kinetic process of crystal growth (cf. Figure 1). Viscosity, in turn, is a function of temperature and composition, and the intersection of the melting curve with the iso-viscosity line yields the point (*w*′, *T*_m_^′^). If freezing is carried out in sufficiently large volumes (consider, e.g., vials at the milliliter scale) where nucleation occurs above *T*_m_^′^, the composition of the solution after nucleation follows the melting line until it approaches a concentration of *w*′ corresponding to the temperature *T*_m_^′^. This process is visualized by the blue line in Figure 4(c).

In the microdroplets studied here, however, nucleation occurs predominantly at temperatures below *T*_m_^′^. In this case (the red line in Figure 4(c)), the freeze-concentrated solution cannot attain the concentration level *w*′, as the viscosity of the hypothetical state (*w*′, *T*^nuc^) is greater than the viscosity at which ice crystal growth is inhibited (see light blue iso-viscosity line). The droplet instead attains the final state (*w*, *T*^nuc^) that corresponds to the viscosity level at which crystal growth is inhibited. Since viscosity increases with decreasing temperature, it must hold that *w*(*T*^nuc^) < *w*′ when *T*^nuc^ < *T*_m_^′^. A smaller sucrose concentration in the freeze-concentrate implies that less water is turned into ice, which may be linked to differences in brightness of the frozen droplets. Naturally, when the droplets are heated, viscosity decreases, and the differences between the droplets vanish as all droplets assume states on the melting line. We use this optical effect in the following section to quantify the value of *T*_m_^′^.

### Freeze-Concentrate and Melting Temperatures

While nucleation is stochastic and each droplet experiences a distinct nucleation event, both *T*_m_^′^ and *T*_m_ are deterministic and therefore experienced simultaneously by all droplets. As a result, to facilitate image analysis, the region of interest for the image analysis was expanded from individual droplets to the columns of the image that contained PFA tubing (the procedure for identifying the tubing is explained in the Methodology). Variability between droplets was confirmed to be less than the accuracy of the thermocouple (0.2 °C), and therefore, the columns of pixels where PFA tubing was present was considered to be a suitable region of interest for quantitative analyses of pixel intensity.

**Figure 5:**
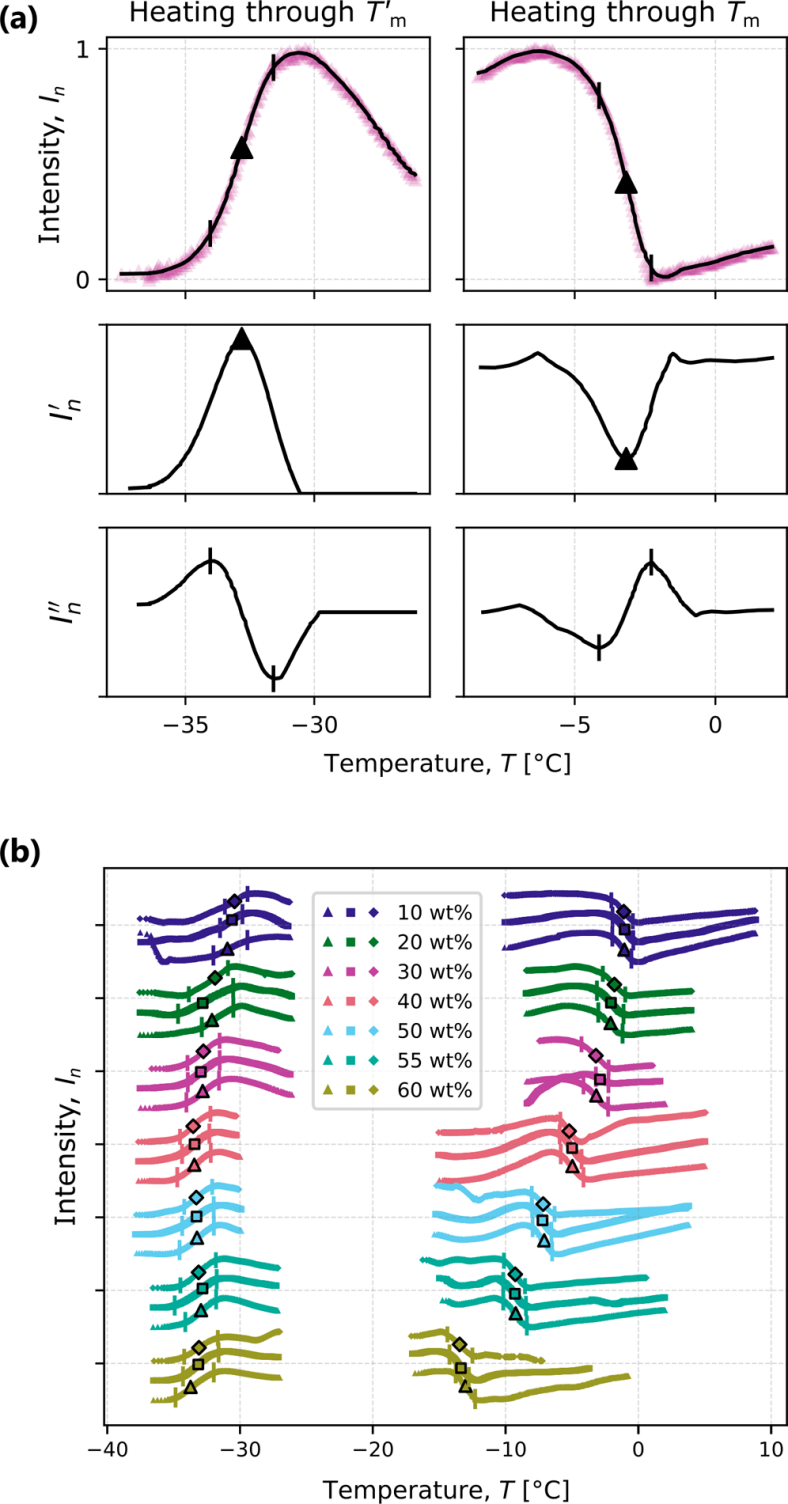
(a) Normalized average column pixel intensity (*I*_*n*_ ∈ [0, 1]) as a function of temperature for the warming (1 °C  min^–1^) portion of the first freeze–thaw cycle of 30 wt % droplets. In the first row, purple triangles are raw data, and the black line is the smoothed data obtained as described in the Methodology section. The midpoint of each transition (denoted by a filled triangle) is defined as the temperature at which the first derivative of intensity (*I*_*n*_^′^, as shown in the second row) reached its maximum (steepest change). Vertical bars denote the temperatures at the onset and endpoint of the transition where the second derivative (*I*_*n*_^′′^, as shown in the third row) reached local extrema before and after the midpoint, respectively. (b) Summary of intensity as a function of temperature for all sucrose concentrations during the warming portions of all experiments. Symbols outlined in black indicate the temperatures at the midpoint of the two transitions, corresponding to *T*_m_^′^ and *T*_m_. For a single experiment, moving upward on the *y*-axis indicates an increase in intensity after cooling, and all experiments are simply offset from each other on the *y*-axis to clearly show the individual trends.

Figure 5(a) illustrates an example of the procedure for identifying important features of the normalized average intensity (*I*_*n*_) of pixel columns as a function of temperature for the warming portion of the first freeze–thaw cycle for 30 wt % sucrose droplets (warming rate of 1 °C/min). As previously shown in Figure 2, heating beyond *T*_m_^′^ is accompanied by an increase in brightness, and heating beyond *T*_m_ by a decrease in brightness. For a quantitative analysis, it is beneficial to consider the derivatives as well: pertinent features of the intensity evolution were extracted from corresponding extrema in the first and second derivatives (*I*_*n*_^′^ and *I*_*n*_^′′^, respectively). The midpoint temperature of the transition was defined as the one where the maximum in the first derivative was reached (shown by the triangle symbols in Figure 5(a)); the onset temperature as the first extremum in the second derivative before the midpoint; and the endpoint temperature as the first extremum in the second derivative after the midpoint. In Figure 5(b), the normalized intensity evolution is shown for all concentrations and all freeze–thaw cycles, with the midpoints identified by symbols outlined in black and onset and endpoint temperatures indicated by vertical segments. To reduce the size of the ensuing data sets, not all images taken during the warming period were saved, but only those around the expected glass transition and melting points, hence leading to gaps in the plotted thermal intensity evolution.

It is worth mentioning that *T*_m_^′^ (but not *T*_m_) may also be obtained by monitoring the droplet brightness after nucleation during the freezing ramp, as demonstrated in Figure 4. We chose the method described here (intensity change upon warming) for the quantitative analysis because it allows for the measurement of both *T*_m_^′^ and *T*_m_.

**Figure 6:**
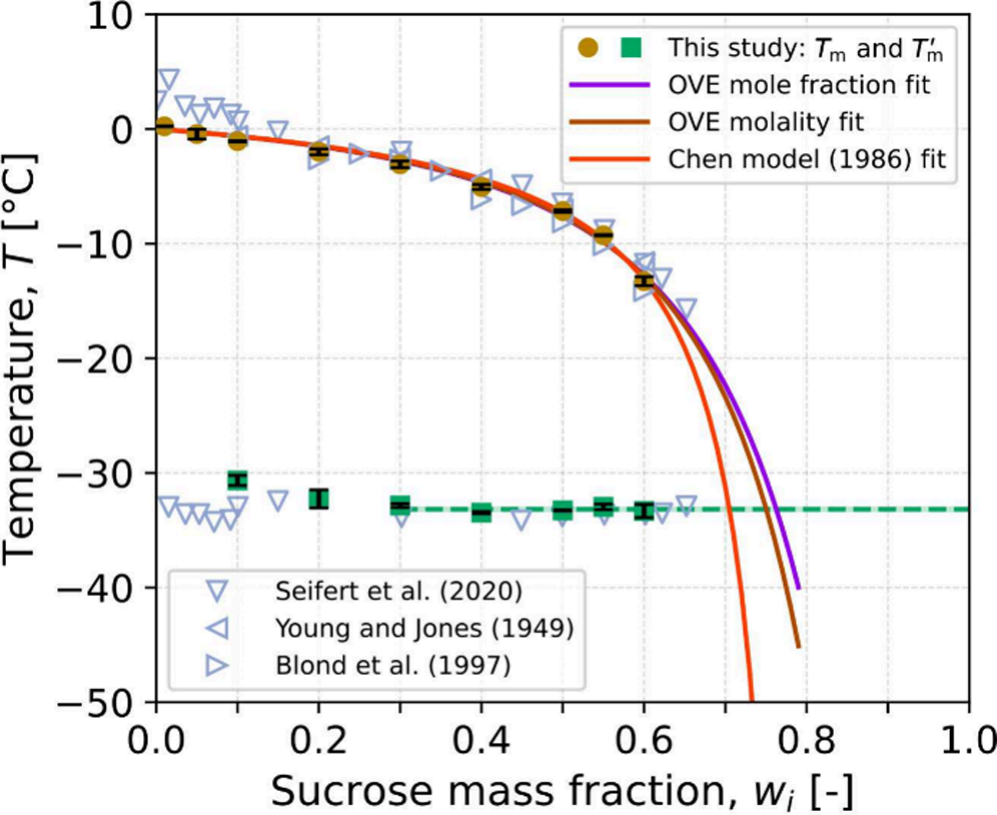
Temperatures in the maximally freeze-concentrated region (*T*_m_^′^) and the melting region (*T*_m_) as a function of sucrose mass fraction. Each gold symbol corresponds to the average temperature over the three cycles for the midpoint of melting (Figure 5), while each green symbol corresponds to the average temperature at the midpoint of the *T*_m_^′^ transition (Figure 5). The error bars show two standard deviations over the three cycles. The horizontal dashed green line depicts this study’s average *T*_m_^′^= −33.2 °C. The solid lines are fits to this study’s *T*_m_ values using two forms of the osmotic virial equation (OVE)^55^ and the Chen model.^56^ Open symbols are experimental measurements of melting point from three literature sources^40,53,54^ and a set of *T*_m_^′^ measurements from Seifert et al.^40^

Figure 6 and Table 1 summarize the observed values for *T*_m_ (gold symbols) and *T*_m_^′^ (green symbols) of sucrose solutions as a function of composition obtained from the analysis of Figure 5. The melting point was defined as the midpoint of the decrease in the pixel intensity averaged over the columns of pixels where tubing was present, and the obtained values are in close agreement with other measurements reported in the literature.^39,53,54^*T*_m_^′^ was taken to be the midpoint of the identified temperature range over which an increase in average pixel intensity was observed. This choice of midpoint is similar to the methodology for interpreting differential scanning calorimetry experiments, where the midpoint temperature of a change in heat flow is taken to be the value of *T*_m_^′^.^27,29^ It can be seen that at the lowest sucrose mass fractions (10–20 wt %), the identified *T*_m_^′^ values are higher than those obtained at higher sucrose mass fractions. This may be attributed to the high nucleation temperatures observed for these solutions (see Figure 3), where a large number of droplets nucleate at or above *T*_m_^′^ and, hence, experience only little or no increase in brightness upon heating. It is worth noting that DSC, the standard method for the measurement of *T*_m_^′^, similarly suffers from weak signals in low-concentrated solutions.^27,29^ In Figure 5, the average grid intensity is seen to slowly increase at a temperature lower than the identified midpoint. This observation could suggest that the onset of the intensity increase may be a more consistent feature to extract *T*_m_^′^ from the intensity evolution at lower sucrose mass fractions. For all concentrations greater than 30 wt %, however, the observed *T*_m_^′^ values are in close agreement within the standard deviation between freeze–thaw cycles at each concentration. Taking the average of *T*_m_^′^ across all concentrations excluding 10 and 20 wt % yields a value of −33.2 ± 0.2 °C, where the uncertainty is one standard deviation. This value is in agreement with the average of −33.5 ± 0.5 °C (one standard deviation) calculated from the values reported by Seifert et al.^40^ over the concentrations studied therein using DSC (shown by upside-down triangles in Figure 6).

**Table 1:** Summary of the Midpoint of the Freeze-Concentrated Glass Transition Temperature (*T*_m_^′^) and the Midpoint Melting Temperature (*T*_m_) as a Function of Sucrose Concentration Obtained from the Data Shown in Figure 5^a^

Sucrose concentration (wt %)	*T*_m_^′^ (°C)	*T*_m_ (°C)
0	—	0.7 ± 0.02
1	—	0.2 ± 0.04
10	–30.7 ± 0.4	–1.1 ± 0.1
20	–32.3 ± 0.8	–2.0 ± 0.3
30	–32.8 ± 0.2	–3.1 ± 0.3
40	–33.5 ± 0.1	–5.1 ± 0.2
50	–33.3 ± 0.04	–7.2 ± 0.1
55	–33.0 ± 0.2	–9.3 ± 0.05
60	–33.3 ± 0.6	–13.3 ± 0.4

a Each value is the average (± two standard deviations) of the three freeze–thaw cycles at that concentration. Thermocouple accuracy is ±0.2 K.^32^

### Concentration of the Freeze-Concentrated Solution

The concentration of sucrose in the maximally freeze-concentrated solution (*w*^′^,*T*_m_^′^) can be obtained by extrapolating the melting line beyond the measured melting points down to the temperature *T*_m_^′^.^27,40^ For fitting the measured melting points, we investigate three theoretical models.

First, we combine the Gibbs–Duhem equation with a model that can describe nonideal solution behavior. We select the osmotic virial equation (OVE) due to its accuracy and rigorous derivation from principles in statistical mechanics,^55,57,58^ as well as for the possibility of using its regressed coefficients to accurately predict properties of solutions with three or more components in relevant applications.^12,55,59^ There are two forms of the osmotic virial equation, yielding two distinct approaches for relating the melting point to the solution composition when each is combined with the Gibbs–Duhem equation. One is based on the osmolality of the solution, π:^55^
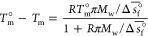
1where *T*_m_^°^ is the melting point of pure water (273.15 K), *R* is the universal gas constant (8.314 J mol^–1^), *M*_w_ is the molar mass of water (18.02 g mol^–1^), and  is the standard molar entropy change of fusion of water (22.00 J mol^–1^ K^–1^). We truncate osmolality, π, to a polynomial of second-order: π = *m*_*i*_ + *B*_*ii*_*m*_*i*_^2^ where *m*_*i*_ is the molality of solute *i* and *B*_*ii*_ is the molality-based second osmotic virial coefficient. The second approach is based on the osmole fraction, π̃:^55^
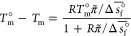
2where we truncate the osmole fraction to a second-order polynomial: , where *x*_*i*_ is the mole fraction and *B*_ii_^*^ is the mole-fraction-based second osmotic virial coefficient. Fitting eq 1 and eq 2 to the melting points reported in Table 1 yields values of *B*_*ii*_ = 0.15 ± 0.01 molal^–1^ (brown line in Figure 6) and *B*_ii_^*^ = 10.6 ± 0.7 (purple line in Figure 6), respectively.

A commonly used semiempirical model in the literature on freeze-drying is the Chen model,^56^ which has the following form:
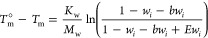
3where *b* is a fitting parameter, *K*_w_ = 1.86 kg K^–1^ mol^–1^ for water, *M*_w_ is the molar mass of water, *w*_*i*_ is the mass fraction of the solute (sucrose), and *E* = *M*_w_/*M*_i_ where *M*_i_ is the molar mass of the solute. Fitting to our data of *T*_m_ in Table 1 yields *b* = 0.30 ± 0.01 and the orange line in Figure 6.

Finally, we set *T*_m_ = *T*_m_^′^ in the obtained fitted models (eqs 1–3) to solve for the corresponding value of *w*′ for the maximally freeze-concentrated solution. From the osmolality-based OVE model, we obtain a value of 75.0 ± 0.1 wt %, and from the osmole-fraction-based OVE, we obtain 76.3 ± 0.1 wt %. Both of these results are in close agreement with literature values that range between 72 wt % and 77 wt %.^60^ On the other hand, the Chen et al. model^56^ fit to our measured data yields a value of 70.5 ± 0.1 wt %. The significant difference between the Gibbs–Duhem–OVE model and the Chen model highlights the sensitivity of the obtained value of *w*′ to the model chosen for extrapolation.

### Ice Crystal Growth

Crystal growth is relevant in the scope of this work for at least two reasons. First, a nucleus upon its formation is extremely small and cannot be detected immediately. The new ice phase requires time to grow to a detectable size; such a delay must be considered when analyzing nucleation data, as is commonly done in studies on nucleation from solution.^61,62^ Second, when interpreting and modeling freezing processes, it is of importance to hypothesize how many nuclei form within the volume of interest. It is typically assumed that a single nucleus initiates growth that encompasses the entire volume before a second nucleus can form (this is plausible if crystal growth is very fast and the volume is small), which is consistent with the description of nucleation as a rare event. Such an assumption is commonly made when analyzing the freezing behavior in microdroplets.^21,33^ It has also been applied in previous studies focusing on larger volumes relevant to pharmaceutical applications,^44,51^ where it was verified through visual observation that freezing in vials starts from a single point of origin, i.e., from a single nucleus.

**Figure 7:**
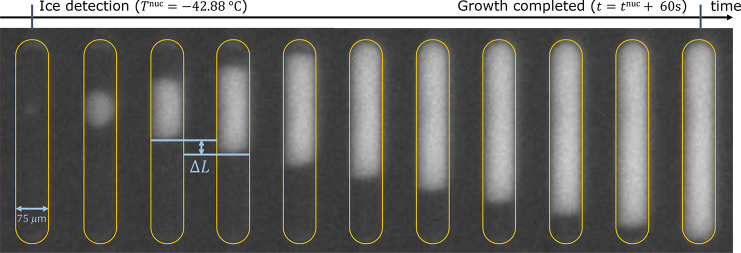
Observation of ice formation in a single slug containing 55 wt % sucrose solution. The slug is about 445 μm long and 75 μm in width, and crystal growth requires about 60 s to encompass its entire volume. A yellow outline representing the extent of complete crystal growth is overlaid at the same position in each frame to facilitate a visual comparison. The nucleation time, *t*^nuc^, is defined as the first point in time, i.e., the first image, where ice is detected; the nucleation temperature, *T*^nuc^, is the temperature measured by the thermocouples at this time.

These previous studies motivated us to carry out a detailed crystal growth analysis. Figure 7 illustrates ice formation in a single slug (75 μm × 445 μm, yellow outline) containing 55 wt % sucrose solution; the use of elongated slugs instead of droplets allows for a monitoring of crystal growth over a longer period of time (see Methods for details on slug generation). The figure consists of a total of 11 cropped images of the same slug, taken every 6 s, representing the evolution of the droplet over a total period of 1 min. The leftmost image is the first image in which ice can be visually detected (bright spot); the rightmost image shows a slug that is nearly completely frozen; i.e., its entire volume appears bright.

Assuming that the maximally freeze-concentrated solution contains 75 wt % sucrose, this phase comprises 73.3% of the slug’s total mass, whereas the ice crystals comprise the remaining 26.7%. Hence, even after ice formation is complete, ice crystals encompass only a minor mass fraction of the slug. Even though the estimated amount of ice crystals is relatively minor, the rightmost image in Figure 7 shows an evenly bright slug indicating the presence of ice throughout its entire volume. This is because there is no macroscopic separation of the two phases, and instead the ice phase and the freeze-concentrate form an intertwined network with contiguous regions having a length-scale on the order of micrometers or below (see e.g., Först et al.^63^ for images of such a crystalline network). The formation of such a network during freezing is in fact the reason why aqueous solutions can be freeze-dried. After the freezing phase of the process is complete, ice crystals sublimate during the primary drying phase under vacuum, leaving behind the highly porous network of the freeze-concentrated phase, which due to its large surface area, allows for a fast desorption of the water in the freeze-concentrate.^15,20^ Given the resolution of 6.8 μm per pixel, it is not possible to observe individual pockets of ice or freeze-concentrate, and instead the entire frozen region appears bright.

An image sequence such as the one shown in Figure 7 can be used to compute the velocity of the freezing front, i.e., of the interface between the region in which ice has already formed and that where it has not. Because crystal growth is the only phenomenon that takes place in the slugs after nucleation (as in most cases only a single nucleus forms per slug), such a velocity represents the crystal growth rate under the given conditions. The growth rate *G* is obtained by measuring the difference in length of the frozen region (Δ*L*) between two images and considering the time elapsed between them (Δ*t*) as *G* = Δ*L*/Δ*t*.

**Figure 8:**
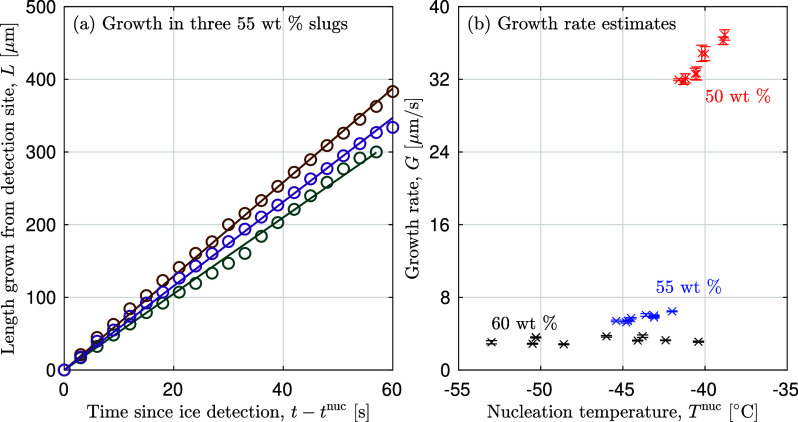
Growth rate estimates for slugs containing 50 wt %, 55 wt %, and 60 wt % sucrose solution. (a) Growth of ice in three slugs containing 55 wt % sucrose solution; they represent the fastest-growing, the slowest-growing, and an average-growing slug out of the ten measured in total. The markers denote experimental measurements, and lines the best fit obtained through linear regression. (b) Growth rate measurements in ten slugs per solution composition, sorted by nucleation temperature. Error bars denote two standard deviations.

The growth rate is a function of the temperature and solution composition. To investigate this relationship, we report in Figure 8 the growth rates measured in ten slugs each for three sucrose concentrations at different temperatures. Panel (a) shows the growth of ice as a function of time in three slugs containing 55 wt % sucrose solution that represent the fastest-growing, the slowest-growing, and an average-growing slug out of the ten measured in total. The markers denote experimental measurements, and lines denote the best fits for growth rate obtained through linear regression. As can be seen, all slugs show a linear evolution of length grown with time, and the growth rate in the fastest-growing slug is about 20% higher than that in the slowest-growing one.

Panel (b) illustrates the regressed values of the growth rates and their uncertainty for all measured slugs, plotted in terms of their nucleation temperature. Average growth rates of 33.4 ± 4.4 μm s^–1^, 5.7 ± 0.8 μm s^–1^, and 3.3 ± 0.6 μm s^–1^ were measured for 50 wt %, 55 wt %, and 60 wt %, respectively. Both the error bars and the uncertainties in the growth rates correspond to two standard deviations. A number of remarks are worth making. First, the growth rate decreases significantly with increasing sucrose concentration. This is because with increasing sucrose concentration the viscosity level of the solution increases and, hence, the molecular mobility of the water molecules decreases. In fact, at concentration levels below 50 wt %, crystal growth was too fast for it to be monitored adequately: the time elapsed between two images suffices for ice to grow into the entire volume of the slug. Hence, this analysis is limited to highly concentrated solutions where crystal growth is slow. Second, at all concentration levels, growth is fast enough that there is no relevant delay in detection of nucleation; i.e., the time to grow to a detectable size is shorter than the time elapsed between two images (3 s). Third, the growth rate decreases with decreasing temperature, particularly for the 50 wt % sucrose solution. The temperature dependence of the growth rate stems from both the thermodynamic driving force (larger at lower temperature) and kinetic effects related to the molecular mobility in the solution (lower at lower temperature). Hence, the growth rate is dominated by kinetic effects for the systems studied here. Finally, all 30 slugs analyzed in this study experienced only one single nucleation event. For 55 wt % and 60 wt % sucrose solutions, however, some slugs not included in the growth rate analysis exhibited a different freezing behavior; that is, two separate frozen regions were observed to form that eventually grew together. Such a scenario is expected for slugs in which two distinct nucleation events take place. It can be explained by considering Figure 7 again, which shows that the time scale for growth in a 55 wt % slug is on the order of one minute. Slugs of this composition nucleate within a temperature interval of about 4 °C, as illustrated in Figure 8(b), corresponding to a time interval of 4 min. Given that both times are on the same order of magnitude, it is indeed statistically reasonable to expect that more than one nucleus will form in some slugs. For the droplets analyzed in the nucleation temperature study, which are 6 times shorter in length than the slugs—and hence experience a 6 times shorter growth time—the single nucleus assumption is reasonable.

To conclude, we compared the estimated growth rates with the literature values. It is worth noting that conventional growth rate experiments are typically carried out by monitoring seeded ice crystals at small supercooling (order of 1 °C), whereas the method presented here naturally operates at large supercooling, namely, at that connected to the stochastic occurrence of nucleation. For example, Blanshard et al.^34^ reported a growth rate of 6.5 ± 0.3 μm s^–1^ for a 58.6 wt % sucrose solution at a temperature of −16.2 °C. This value is of the same order of magnitude as those measured here for the 55 wt % and 60 wt % sucrose solutions, and it agrees well with the observation of a weak temperature-dependence. Since growth rates can be measured at relatively low temperature (high supercooling), the method presented here promises to complement existing techniques applicable to higher temperatures (lower supercooling). For high supercooling levels, growth rates for droplets of pure water have been reported by Schremb et al.,^64^ which are significantly higher, namely on the order of 15 cm s^–1^ for a supercooling of 20 °C. The same study reported an increase in crystal growth rate with increasing supercooling, i.e., the opposite trend that we observed here for concentrated sucrose solutions. This is not surprising, given the significant differences in viscosity and hence molecular mobility between pure water and concentrated sucrose solution.

## Conclusions

The importance of phase equilibrium predictions and of the kinetics of phase transitions is evident in the design and control of freezing and freeze-drying processes. Motivated by this, we investigated and demonstrated the use of droplet microfluidics to aid in mapping out the solid–liquid phase boundaries and the associated kinetics for a sucrose–water system at concentrations below the eutectic point. As a function of sucrose composition, three key temperatures were extracted based on an analysis of temporal changes in pixel intensity: the nucleation temperature distribution, the melting temperature (*T*_m_), and the temperature of the maximally freeze-concentrated solution at (*w*′, *T*_m_^′^). Knowledge of the last two enabled the computation of the sucrose concentration in the maximally freeze-concentrated solution, another important design parameter for biopharmaceutical formulations.

Additionally, slugs (elongated droplets) were generated in separate experiments comprising highly concentrated sucrose solutions, and the growth rate of ice crystals was quantified to yield insights into its dependence on both composition and temperature. The growth rate was observed to decrease at higher concentrations and lower temperatures, likely due to the reduction in molecular mobility. Further, we assessed the commonly made assumption that a single nucleation event occurs, confirming that it is valid for spherical droplets, while occasionally two nuclei were observed for the slugs with the highest sucrose concentration.

Overall, we showed the ability of droplet microfluidics to characterize and quantify the freezing behavior of aqueous sucrose solutions, both thermodynamically and kinetically. Future work can be pursued for other mixtures of interest to the broad range of applications in which controlling or understanding freezing is relevant—in industry (food, pharmaceutics, and cryobiology) and in the environment (the atmosphere).

## Methodology

### Experimental Methods

The Microfluidic Ice Nuclei Counter Zurich (MINCZ) was used to generate and control the temperature of monodisperse populations of 75-μm droplets. The operating principle of MINCZ is described in detail by Isenrich et al.^32^ First, microchannels were patterned onto an SU-8 coated silicon wafer, followed by standard soft lithography to transfer the channels to a polydimethylsiloxane (PDMS, Elastosil RT 601 A/B, Ameba AG, Switzerland; mass ratio of 10:1 between the base and curing agent) device bonded to a glass slide (Menzler-Glaser, Germany) via plasma treatment. Second, immediately prior to droplet generation, a fresh sucrose solution of the desired concentration was prepared. Solution preparation entailed: (i) cleaning glassware with deionized water (Millipore, Milli-Q Advantage A10 system), acetone, and an additional three times with deionized water; (ii) weighing the desired mass of sucrose (Sigma-Aldrich, BioXtra grade, >99.5% purity) and fully dissolving it in deionized water for a total solution mass of 50 g; (iii) filtering the sucrose solution (0.22 μm hydrophilic PTFE syringe filter); and (iv) transferring the solution to a glass vial (Lab Logistics Group GmbH, 1.5 mL) using a 100–1000 μL pipet (Socorex Acura 825). Third, to generate microfluidic droplets, three glass syringes (1 mL, Hamilton^Ⓡ^ syringe, Sigma-Aldrich) were placed in syringe pumps (Aladdin AL1000-220Z, World Precision Instruments, USA) to control the flow rates of the sucrose solution, fluorosurfactant (2% v/v 008-FluoroSurfactant in HFE-7500 (RAN Biotechnologies, USA)), and fluorinated HFE-7500 oil (3M Novec 7500, Interelec Electronics AG, Switzerland) into the microfluidic device. Depending on the target droplet size, a different channel geometry was used: for small droplets of approximately 75 μm in diameter, the same channel geometry as described in Isenrich et al.;^32^ and for elongated droplets (slugs), a T-junction channel geometry. Generated droplets exited the microfluidic device through an outlet connected to high-purity perfluoroalkoxy alkane (PFA) tubing (up to 50 cm in length; 360 μm o.d., 75 μm i.d.; IDEX Health & Science LLC, USA) held in a custom-milled polyether ether ketone (PEEK) structure. After droplets were generated, the PFA tubing was cut at the outlet of the microfluidic device with scissors, and the ends of the tubing were mechanically clogged with serrated forceps. Finally, the PFA tubing was placed in an ethanol bath, the temperature of which was regulated using a Peltier element (PKE 128A 0020 HR 150, Peltron GmbH, Germany) and recirculating chiller (Huber KISS K6, Huber Kältemaschinenbau AG, Germany) with a working fluid of aqueous 55% v/v ethylene glycol (98% technical grade, Sigma-Aldrich, USA). The polarity of the Peltier element was set by an Arduino UNO R3 (Arduino) with two single-pole double-throw (SPDT) switches (Grove 2-Channel SPDT Relay, Seeed Technology Co., Ltd.) wired to create a double-pole double-throw (DPDT) switch. Temperature was measured with two K-type thermocouples (0.5 mm o.d., RS Components GmbH, Germany, and TC Direct, Germany) placed horizontally in the same plane as the droplets (see Isenrich et al.^32^ and Shardt et al.^33^ for more details). During both droplet generation and cooling, a stereoscope (Nikon SMZ1270 (0.5× objective lens) equipped with a fiber ring illuminator with LED light source) and CMOS camera (iDS UI-3060CP-M-GL Rev. 2) were used to observe the droplets.

Aqueous sucrose solutions with concentration levels of 0 wt %, 1 wt %, 10 wt %, 20 wt %, 30 wt %, 40 wt %, 50 wt %, 55 wt %, and 60 wt % were studied with monodisperse droplet populations. Each droplet population underwent three freeze–thaw cycles that traversed three temperature regions of interest: the nucleation, maximally freeze-concentrated glass transition, and melting temperatures. The cycles were implemented with the recirculating chiller at either: (i) a constant set point temperature of −17 °C with the Peltier element controlling the ethanol bath temperature (necessitating a reversal of the Peltier element’s polarity to reach the solutions’ melting temperatures) or (ii) a dynamic temperature set point with an unchanged polarity for the Peltier element. A constant chiller temperature permits experiments to rapidly scan through the temperatures of interest. On the other hand, a dynamic chiller temperature permits constant cooling and warming rates to be maintained for all temperatures. Droplet size and cooling rate were selected to match the conditions used in an earlier study that focused on the monitoring of homogeneous ice nucleation in water droplets.^33^

### Image Analysis

Nucleation, maximal freeze-concentration, and melting temperatures were identified based on changes in the pixel intensity of the region of interest (implemented with OpenCV and SciPy in Python). For nucleation temperatures, the region of interest was each individual droplet, because nucleation is a stochastic process and each droplet nucleates at a different temperature. For the maximally freeze-concentrated and melting temperatures, the regions of interest were the columns of pixels where PFA tubing was present because these processes are deterministic, thus lending themselves to a simplified image processing approach.

#### Identifying Which Pixels Contain Tubing

To reduce the computational time for image processing, only the regions of each image that contained tubing were used for further image analysis. These regions were found with the following procedure performed on the first image saved in the experiment: equalizing the histogram, applying Otsu’s thresholding, calculating the mean pixel value of each pixel column in the image, smoothing the mean pixel value with a Savitzky–Golay filter, and identifying the regions with peaks in pixel intensity. The identified peaks in the pixel intensity corresponded to the presence of a piece of tubing in the image.

#### Droplet Nucleation Temperature, *T*^nuc^

Due to the stochasticity of nucleation, the average intensity of each droplet was tracked. To determine the droplets’ locations, the following procedure was followed. The last image in the saved sequence was binarized, and then morphological opening was applied to remove extraneous bright pixels. The Hough circle transform was applied to find circular shapes (i.e., the droplets), and the average intensity of a 9-pixel radius circle at the identified center coordinate was calculated for each saved image.

To determine the temperature at which a droplet increased in brightness, the observed temporal evolution of the droplet intensity was analyzed. A Savitzky–Golay filter was applied to smooth the time series of average intensity, and the first derivative of intensity was calculated with respect to time for each consecutive pair of images. The temperature at which the first derivative reached its maximum was taken to be the temperature at the midpoint of the transition between the liquid and the solid. Next, the second derivative was calculated, and the temperatures where the second derivative reached extrema were identified (corresponding to the beginning and end of the phase transition, respectively). In Figure 3, the temperature plotted on the *x*-axis is the one at the beginning of the transition. The results were reviewed manually to remove from consideration any droplets that were exceptionally large or had merged between freeze–thaw cycles.

#### *T*_m_^′^ and *T*_m_

The average pixel value of the regions with tubing was calculated for each image. For both *T*_m_^′^ and *T*_m_, the first and second derivatives were calculated. A maximum in the absolute value of the first derivative was assigned as the midpoint of the transition in brightness. The extrema in the second derivative corresponded to the beginning and end of the transition regions. An increase in pixel intensity was observed as the temperature increased above the maximally freeze-concentrated temperature, while a decrease in pixel intensity was observed during melting (the solid-to-liquid phase transition).

#### Slug Generation and Crystal Growth Rate

For each experiment, ten slugs were selected for growth rate measurement based on the following criteria: (i) only a single nucleus formed in the slugs, (ii) the nucleus forms close to the top or bottom to allow for more time until growth is complete, and (iii) the set of slugs represents the entire range of nucleation temperature. The grown length was measured in all images after nucleation based on the pixel intensity profile. A single pixel corresponded to a distance of 6.8 μm.

## Data Availability

All plotted figure data can be found at https://doi.org/10.3929/ethz-b-000664062.
